# Modeling the effect of a genetic factor for a complex trait in a simulated population

**DOI:** 10.1186/1471-2156-6-S1-S87

**Published:** 2005-12-30

**Authors:** Mathieu Bourgey, Anne-Louise Leutenegger, Emmanuelle Cousin, Catherine Bourgain, Marie-Claude Babron, Françoise Clerget-Darpoux

**Affiliations:** 1 INSERM Unité 535, B.P. 1000, 94817 Villejuif Cedex, France, Villejuif, France; 2 INSERM U679, Paris, France; 3 Evry Genetics Center, Aventis Pharma, Evry, France

## Abstract

Genetic Analysis Workshop 14 simulated data have been analyzed with MASC(marker association segregation chi-squares) in which we implemented a bootstrap procedure to provide the variation intervals of parameter estimates. We model here the effect of a genetic factor, S, for Kofendrerd Personality Disorder in the region of the marker C03R0281 for the Aipotu population. The goodness of fit of several genetic models with two alleles for one locus has been tested. The data are not compatible with a direct effect of a single-nucleotide polymorphism (SNP) (SNP 16, 17, 18, 19 of pack 153) in the region. Therefore, we can conclude that the functional polymorphism has not been typed and is in linkage disequilibrium with the four studied SNPs. We obtained very large variation intervals both of the disease allele frequency and the degree of dominance. The uncertainty of the model parameters can be explained first, by the method used, which models marginal effects when the disease is due to complex interactions, second, by the presence of different sub-criteria used for the diagnosis that are not determined by S in the same way, and third, by the fact that the segregation of the disease in the families was not taken into account. However, we could not find any model that could explain the familial segregation of the trait, namely the higher proportion of affected parents than affected sibs.

## Background

The aim of this work is to study and model the marginal effect of one susceptibility locus involved in the determinism of Kofendrerd Personality Disorder (KPD) in the Aipotu population. The presence of a susceptibility locus closely linked to the marker C03R0281 was shown by the existence of both strong association and genetic linkage between this marker and the trait [1,2]. Before modeling the marginal effect of this factor, we searched for the replicate that best represented this effect. The modeling (estimation of the allele frequency and marginal penetrances) is carried out through the MASC method [3], using the information provided by the marker C03R0281 denoted M hereafter. The variation intervals for the parameter estimates are obtained through a bootstrap procedure we incorporated in the MASC (marker association segregation chi-squares) program.

## Methods

### Selection of the best replicate

We want to select the replicates that best represent the distributions in the region of marker M (C03R0281) in terms of both association and linkage. Estimating the parameters and their variation intervals in this sample is then equivalent to evaluating them in the whole set of replicates. In the pooled sample set (10,000 families), we consider one index (an affected case) by family and his genotype for the marker M. For each index, we also consider his identity-by-descent (IBD) sharing for M with one random affected sib (families of the Aipotu population have been selected as having at least two sibs affected with KPD). In order to have a reliable IBD sharing for each sib pair, we ordered one SNP packet (153) surrounding marker M. The IBD sharing is obtained by maximum likelihood estimation using the information provided by the whole set of markers in the M region on chromosome 3.

**Figure 1:**
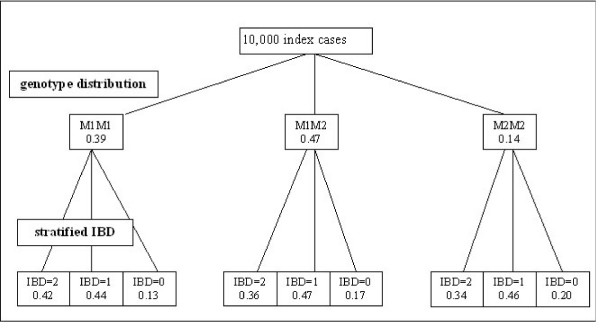
**Distribution of the marker C03R0281 for the 10,000 index cases of the Aipotu population**. The first level shows the genotype distribution of the 10,000 index cases for the marker M. The second level shows, for each marker genotype, the index cases classified according to their IBD sharing with one affected sib (stratified IBD distribution).

Figure 1 gives the genotype distribution of the 10,000 index cases for the marker M. The two alleles of M, M1 and M2, have a frequency of 0.56 and 0.44, respectively. For each marker genotype, the index cases are classified according to their IBD sharing with one affected sib (stratified IBD distribution).

Because we are looking for the model that best explains these four independent distributions (one genotype distribution and three stratified IBD distributions), we first determined the replicates that best reflect these distributions. We computed the distance of each of the 100 replicates to the pooled sample by a chi-square statistics, equal to the sum of the four independent chi-squares obtained by comparing the distributions observed in the replicate and in the pooled sample.

### Modeling the genetic effect

We modeled the effect of the susceptibility factor by the MASC method. For a given genetic model, the MASC method computes the four expected distributions described in Figure 1 and the previously described chi-square statistics (the sum of four chi-squares between the observed and expected distributions). The chi-square is minimized over the parameters left free to vary. The fit of the model to the observed data is then tested (8 df minus the number of parameters free to vary). The parameters of the genetic model are the penetrances of each genotype and the coupling between the marker alleles and the susceptibility factor alleles. The expected distributions are computed conditionally to the fact that the index cases have at least one affected sib. They depend on the frequency of the marker alleles in the general population. The marker allele frequencies may be assumed to be already known (situation 1), to be obtained through a control sample (situation 2), or to be obtained through the parental alleles which have not been transmitted to the affected cases used for the family ascertainment (situation 3).

### Computing the intervals of variation for the parameter estimates

We implemented a bootstrap procedure for calculating the variation intervals of the parameter estimates in the MASC program. The uncertainty on the parameters is due to the sampling of families and of controls, when the marker allele frequencies are inferred from a control sample. For each bootstrapped family set (1,000 replicates), we estimated the parameters considering the three possibilities described above for the marker allele frequencies. In situation 1, there is no uncertainty induced by the marker allele frequencies. In situation 2, the bootstrap procedure is applied to both the family sample and a sample of 50 controls randomly drawn among the 100 control samples. In situation 3, the bootstrap is only applied to the family sample. For the three situations, we obtain the distribution of the parameter estimates and provide the 95% intervals.

## Results

### The best replicate

Table 1 gives the ten replicates that best represent the distributions in the marker M area (the smallest chi-squares). Replicate 97 was chosen for the following analyses.

### Modeling of the genetic effects

We tested a biallelic susceptibility locus model (S1, S2) and estimate four parameters: 2 relative penetrances, λ1 and λ2, and 2 coupling probabilities, c_11_and c_12_, where

λ1 = P(affected | S1S2) / P(affected | S1S1),

λ2 = P(affected | S2S2) / P(affected | S1S1),

c_11 _= P(S1 | M1),

c_12 _= P(S1 | M2).

The frequency (q) of allele S1 at the susceptibility locus can be written as

q = P(S1) = c_11 _P(M1) + c_12 _P(M2).

The direct effect of marker M, which means S1 is confounded with M1, and S2 with M2, was rejected (χ^2 ^= 14.14; 6 df).

However, many biallelic models are not rejected. We give several models compatible with the observations made on M in replicate 97 (Table 2). To discriminate between these models, we looked for those that also fit the observations on the closely linked markers. Among these markers, three in packet 153 were also associated with KPD: SNPs 16, 17, and 18 (results shown in Table 3). The direct effect of SNP 16, 17, or 18 was also rejected and these three SNPs were not significantly in linkage disequilibrium (LD) with each other or with marker M (SNP 19). We therefore concluded that the functional polymorphism has not been typed and is in LD with the four studied SNPs.

### Computing the variation intervals of the parameter estimates

Table 4 gives the 95% variation intervals of the disease allele frequency q. The intervals are given assuming uncertainty or not on the allele frequencies of marker M. Because the results are very close using a sample of 50 controls or the untransmitted alleles of the 100 families, only results for the latter situation are given in Table 4. In order to show the effect of the family sample size on the variation intervals, we also give the results obtained on the family sample resulting from pooling the two best replicates (97 and 63; Table 1) and from pooling the five best replicates (97, 63, 19, 48 and 56; Table 1). We show that the estimates at the susceptibility allele frequency decrease with the number of families (0.24 per 1 replicate; 0.20 per 5 replicates). However, the uncertainty on the disease allele frequency estimate is very large. The size of the variation interval decreases when the sample size increases mainly by the upper limit. Expected for the largest sample size (500 families), the size of the variation interval does not depend on whether the uncertainty on the marker allele frequencies is taken into account or not.

## Discussion

### Before knowing the simulation model

We have modeled one susceptibility locus S for KPD using the diagnosis criteria of the Aipotu population. It is very likely that the different sub-criteria used for this diagnosis are not determined by S in the same way. The distribution of the sub-phenotypes in all the affected and all the unaffected individuals as well as the IBD distribution between affected sibs in the pooled set of 10,000 families is given in Table 5. All affected individuals display the sub-phenotype combination (e + f + h) compared to only 4‰ of unaffected individuals. This means that the diagnostic criteria in Aipotu are equivalent to having simultaneously sub-phenotypes (e + f + h). There is no distortion in the IBD distribution of sub-phenotypes i, j, and l. In addition, i and j have the same frequency in affected and unaffected individuals. The sub-phenotype k shows the strongest IBD distribution distortion (0.1, 0.49, 0.41 for IBD = 0, 1, 2, respectively).

The observed distributions do not provide any information on the dominant parameter of the disease locus. Indeed, the 95% variation interval of λ1 ranges from 0 to 1. Information can however be improved by taking into account the familial recurrence risk for KPD. The proportion of affected parents is 0.2 and the proportion of affected sibs of indexes is 0.1, after excluding the two sibs by which the families were ascertained (Table 6). The recurrence risk is thus twice as high in parent than in sibs, which cannot be explained by different genetic models, except different penetrances between the generations.

### After knowing the simulation model

To validate our bootstrap procedure, we looked to see if the true parameters used for the simulation were included in our variation intervals. The value of the disease allele frequency used in the simulation is 0.15. This value is included in the variation intervals for the three sample sizes we used (100, 200, and 500 families). The larger the sample size, the closer the estimate to the true value.

Note that the true value of the dominance parameter cannot be inferred from the provided answers without extensive work. Indeed, the KPD phenotype is a mixture of different phenotypes, each one corresponding to different models of interaction between D2 and another susceptibility locus. Because there is no generation effect in the simulation, we still cannot explain the greater risk for parents than for sibs.

## Abbreviations

GAW14: Genetic Analysis Workshop 14

IBD: Identity by descent

KPD: Kofendrerd Personality Disorder

LD: Linkage disequilibrium

MASC: Marker association segregation chi-squares

SNP: Single-nucleotide polymorphism

## Authors' contributions

MB performed the MASC and bootstrap analyses and drafted the manuscript. A-LL carried out the selection of the SNPs and draft the manuscript. EC carried out the study of the LD in the region, CB the association studies, M-CB the linkage studies. FC-D conceived, designed, coordinated the study and helped to draft the manuscript.

## Figures and Tables

**Table 1:** The 10 best replicates. Chi-squares between the distributions observed in the replicate and in the pooled sample.

Rank	Replicate number	*χ*^2 ^value
1	97	0.53
2	63	1.12
3	19	1.15
4	48	1.26
5	56	1.38
6	4	1.39
7	31	1.77
8	55	1.90
9	88	1.91
10	5	1.93

**Table 2:** Models compatible with observations made on M in replicate 97

	λ1	λ2	Q	χ^2 ^(df)
General	0.02	0.001	0.002	1.852 (4 df)
Dominant	1	0	0.001	5.468 (6 df)
Recessive	0	0	0.249	4.005 (6 df)

**Table 3:** Frequencies of allele 1 for SNPs 16, 17, 18, and 19 for the index cases and the controls

SNP	Index cases (*n *= 10,000)	Controls (*n *= 5,000)	*χ*^2 ^(1 df)
16	0.70	0.50	580.84
17	0.40	0.27	258.40
18	0.67	0.54	225.47
19 (marker M)	0.62	0.55	73.61

**Table 4:** Variation interval of the disease allele frequency q

	95% Variation interval [range]^a^	
	
No. Families	No uncertainty	Uncertainty
100	0.24 [0.01; 0.74]	0.24 [0.01; 0.74]
200	0.22 [0.01; 0.54]	0.22 [0.01; 0.55]
500	0.20 [0.07; 0.35]	0.20 [0.01; 0.35]

^a ^95% variation interval of the disease allele frequency q when the marker allele frequencies are known ("no uncertainty") and when they are estimated from the untransmitted parental alleles ("uncertainty").

**Table 5:** Phenotype distribution for the Aipotu population

	A					B				C		
			
	a	b	c	d	e	f	g	h	i	J	k	l
Aff %	0.16	0.67	0.63	0.63	1	1	0.63	1	0.15	0.15	0.44	0.13
Unaff %	0.02	0.04	0.1	0.1	0.1	0.09	0.1	0.1	0.15	0.15	0.09	0.05
IBD = 0	0.19	0.17	0.19	0.2	0.16	0.16	0.2	0.16	0.24	0.25	0.1	0.25
IBD = 1	0.51	0.49	0.47	0.47	0.46	0.46	0.46	0.46	0.49	0.49	0.49	0.48
IBD = 2	0.30	0.34	0.34	0.32	0.38	0.38	0.34	0.38	0.27	0.25	0.41	0.27

**Table 6:** Proportion of affected parents and sibs

	Proportion of affected subjects (*n*/total)	
	
Replicate	Parents	Sibs*
		
All	0.2 (4043/20000)	0.1 (2882/28174)
Best replicate (97)	0.2 (40/200)	0.06 (17/281)

*without the two affecteds used for the family ascertainment
